# Enhancing Breast Lesion Detection in Mammograms via Transfer Learning

**DOI:** 10.3390/jimaging11090314

**Published:** 2025-09-13

**Authors:** Beibit Abdikenov, Dimash Rakishev, Yerzhan Orazayev, Tomiris Zhaksylyk

**Affiliations:** Science and Innovation Center “Artificial Intelligence”, Astana IT University, Astana 010000, Kazakhstan; y.orazayev@astanait.edu.kz (Y.O.); zhaksylyk.tomiris@astanait.edu.kz (T.Z.)

**Keywords:** mammography, deep learning, lesion detection, transfer learning, data augmentation, preprocessing, object detection, YOLO, Cascade R-CNN, RTMDet, RT-DETR, transformer

## Abstract

Early detection of breast cancer via mammography enhances patient survival rates, prompting this study to assess object detection models—Cascade R-CNN, YOLOv12 (S, L, and X variants), RTMDet-X, and RT-DETR-X—for detecting masses and calcifications across four public datasets (INbreast, CBIS-DDSM, VinDr-Mammo, and EMBED). The evaluation employs a standardized preprocessing approach (CLAHE, cropping) and augmentation (rotations, scaling), with transfer learning tested by training on combined datasets (e.g., INbreast + CBIS-DDSM) and validating on held-out sets (e.g., VinDr-Mammo). Performance is measured using precision, recall, mean Average Precision at IoU 0.5 (${\mathrm{mAP}}_{50}$), and F1-score. YOLOv12-L excels in mass detection with an ${\mathrm{mAP}}_{50}$ of 0.963 and F1-score up to 0.917 on INbreast, while RTMDet-X achieves an ${\mathrm{mAP}}_{50}$ of 0.697 on combined datasets with transfer learning. Preprocessing improves ${\mathrm{mAP}}_{50}$ by up to 0.209, and transfer learning elevates INbreast performance to an ${\mathrm{mAP}}_{50}$ of 0.995, though it incurs 5–11% drops on CBIS-DDSM (0.566 to 0.447) and VinDr-Mammo (0.59 to 0.5) due to domain shifts. EMBED yields a low ${\mathrm{mAP}}_{50}$ of 0.306 due to label inconsistencies, and calcification detection remains weak (${\mathrm{mAP}}_{50}$ < 0.116), highlighting the value of high-capacity models, preprocessing, and augmentation for mass detection while identifying calcification detection and domain adaptation as key areas for future investigation.

## 1. Introduction

Breast cancer is one of the most prevalent cancers globally and the leading cause of cancer-related mortality among women. Early detection through mammography screening significantly improves patient outcomes by enabling timely intervention [1]. Digital mammography, the most widely used imaging modality for breast cancer detection, allows for the identification of suspicious lesions such as masses and microcalcifications [2]. Recent advancements in deep learning have revolutionized computer-aided detection (CAD) systems, offering unprecedented accuracy in lesion detection. State-of-the-art object detection architectures, including the YOLO family [3], Cascade R-CNN [4], RTMDet [5], and transformer-based models like DETR [6], have shown strong performance on natural image benchmarks (e.g., RTMDet-x with mean Average Precision (mAP) scores of 52.8 on COCO [7]), prompting their adaptation to medical imaging tasks.

However, mammography presents unique challenges, including subtle lesions, high-resolution images, and variability across datasets due to differences in imaging devices, patient populations, and annotation schemes, which introduce domain shifts that hinder model generalization. To address these challenges, several large-scale mammography datasets have been made publicly available, such as Curated Breast Imaging Subset of DDSM (CBIS-DDSM) [2], VinDr-Mammo [8], the EMory BrEast Imaging Dataset (EMBED) [9], and INbreast [10]. These datasets provide a wealth of annotated images for training and evaluating CAD systems but also require robust methods to generalize across differing data characteristics.

In this work, we propose the following key contributions to advance mammogram lesion detection using deep learning:

- Systematic evaluation of advanced deep learning object detectors, including Cascade R-CNN, YOLOv12 (S, M, L, and X variants), RTMDet-X, and RT-DETR-X, on four mammography datasets (CBIS-DDSM, VinDr-Mammo, EMBED, and INbreast) for detecting masses and calcifications.
- Standardized preprocessing pipeline with contrast-limited adaptive histogram equalization (CLAHE), breast cropping, and data augmentation techniques like rotations and scaling to enhance model robustness.
- Transfer learning assessment by fine-tuning pretrained models on dataset pairs (e.g., CBIS-DDSM+VinDr-Mammo) and testing on held-out datasets (e.g., INbreast), leveraging additional medical imaging datasets (e.g., VinDr-CXR) to improve generalization.

Our results demonstrate that larger YOLOv12 models, particularly YOLOv12-X, and Cascade R-CNN deliver superior performance, achieving mean Average Precision (${\mathrm{mAP}}_{50}$) scores up to 0.963 on INbreast. Transfer learning experiments show that training on multiple datasets enhances model generalization across varying imaging conditions. However, a noticeable performance drop occurs when evaluating on held-out datasets, indicating persistent domain shifts due to differences in imaging devices, patient demographics, and annotation protocols. These findings highlight the critical role of meticulous preprocessing including CLAHE and breast cropping, combined with robust data augmentation strategies like rotations and scaling, in improving model performance for mammogram analysis. Moreover, this study underscores the potential of advanced deep learning detectors to generalize effectively in medical imaging despite challenges posed by dataset variability. By leveraging transfer learning and additional datasets like VinDr-CXR, we mitigate some domain shift effects, but further research is needed to fully address these challenges. This work provides valuable insights into optimizing deep learning models for breast cancer detection, emphasizing the need for standardized preprocessing and robust training strategies to ensure reliable performance across diverse mammography datasets.

## 2. Related Work

The application of deep learning to mammography has seen significant advancements, with various architectures being adapted for lesion detection. Early computer-aided detection (CAD) systems relied on handcrafted features, but deep convolutional neural networks (CNNs) have since demonstrated superior performance. For instance, Ribli et al. utilized RetinaNet for detecting masses and calcifications, while other studies have applied Faster R-CNN and YOLO to this task [11]. The literature on deep learning for mammography lesion detection is extensive and continues to evolve. Below, we summarize key contributions from recent studies, focusing on major approaches and their relevance to our work.

### 2.1. YOLO-Based Methods

The YOLO (You Only Look Once) family of detectors has been particularly effective due to its balance of speed and accuracy. Al-Masni et al. (2018) introduced a YOLO-based CAD system for simultaneous detection and classification of breast masses [12]. Subsequent research has introduced variants such as Zhang et al.’s (2022) anchor-free YOLOv3 for mass detection [13] and Aly et al.’s (2021) optimized YOLOv3 with better anchors [14]. Baccouche et al. (2021) developed a YOLO-based fusion model for multi-view lesion detection [15], while Su et al. (2022) integrated YOLOv5 with transformer-based segmentation for comprehensive lesion analysis [16]. More recently, YOLOv12 has emerged as a cutting-edge model, though its application to mammography remains limited. These studies highlight the adaptability of YOLO models to mammography tasks, emphasizing their efficiency and robustness.

### 2.2. Transformer-Based Methods

Transformer-based architectures have gained traction for their ability to capture global dependencies in images. Chen et al. (2022) used a Vision Transformer to process four mammographic views simultaneously, achieving improved diagnostic accuracy [17]. Betancourt et al. (2023) employed a Swin Transformer for mass detection, leveraging its ability to capture long-range dependencies [18]. Kamran et al. (2023) introduced Swin-SFTNet, a transformer-based U-Net for segmenting micro-lesions in mammograms [19]. While DETR and its variants, such as RT-DETR-X, have shown promise in general object detection, their application to mammography remains underexplored [20]. These advancements underscore the potential of transformers in handling complex mammography data.

### 2.3. Unsupervised and Ensemble Methods

Multi-view fusion strategies, such as Merged Dual-View and Dual-Branch Ensemble models, have further improved detection accuracy in mammography [21]. Unsupervised learning methods have been explored to address the scarcity of labeled data in medical imaging. Park et al. (2023) used StyleGAN2 for unsupervised anomaly localization in mammography, demonstrating the potential of generative models [22]. Ensemble and fusion techniques have also been employed to boost detection performance. McKinney et al. (2020) achieved radiologist-level performance by combining Faster R-CNN with additional CNN classifiers [23], while Manali et al. (2024) proposed a three-channel system integrating support vector machines (SVMs) and CNNs with decision fusion for robust lesion detection [24]. These approaches highlight innovative strategies to enhance detection accuracy in data-constrained scenarios.

### 2.4. Transfer Learning

Transfer learning is crucial for adapting models trained on large natural image datasets to medical imaging tasks. Pan and Yang (2010) provide a foundational survey of transfer learning techniques, widely applied in medical imaging to leverage knowledge from diverse domains [25]. In mammography, transfer learning enables models to generalize across datasets with varying imaging characteristics and annotation schemes. However, domain shifts remain a challenge, as highlighted by Agarwal et al. (2020), who benchmarked Faster R-CNN on the OPTIMAM database [26]. These studies emphasize the need for robust transfer strategies to address domain variability.

### 2.5. Comprehensive Overviews

Recent reviews, such as Carriero et al. (2024), provide a comprehensive overview of deep learning advancements in breast cancer imaging, summarizing state-of-the-art techniques as of early 2024 [27]. These reviews highlight the growing adoption of transformer-based models and the need for standardized evaluation protocols to ensure consistent performance assessment across studies.

Our study builds on these advancements by evaluating cutting-edge models like YOLOv12 and RT-DETR-X, which are not yet extensively explored in mammography. We compare Cascade R-CNN, YOLOv12 variants, and RT-DETR-X across multiple mammography datasets, focusing on preprocessing, augmentation, and cross-dataset transfer learning to address domain shifts and enhance generalization.

## 3. Materials and Methods

### 3.1. Datasets

**Figure 1:**
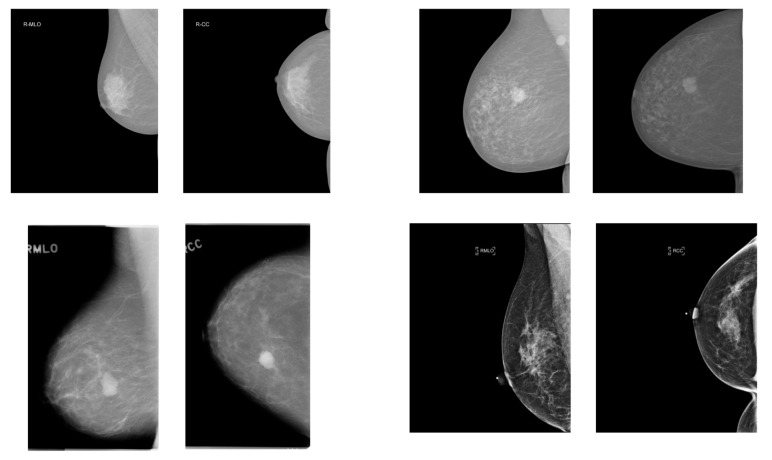
Examples of craniocaudal (CC) and mediolateral oblique (MLO) view pairs from mammography datasets. (**top left**): VinDr-Mammo, (**top right**): INbreast, (**bottom left**): CBIS-DDSM, (**bottom right**): EMBED. In each pair, the MLO view is shown on the left and the CC view on the right.

We used four public mammography datasets. The summary of the datasets is provided in Table 1. Masses and calcifications were chosen as the target lesions due to their high clinical relevance in breast cancer screening, as they are the most common abnormalities detected in mammography and are consistently annotated across all four datasets. In addition, example images are provided in Figure 1.

*INbreast* comprises 115 patient cases (410 images) of FFDM studies collected from the Breast Centre at Centro Hospitalar de S. João (CHSJ), Porto, Portugal, between April 2008 and July 2010. In this dataset, 90 of these cases include four views (both breasts), and 25 cases include two views (post-mastectomy). INbreast contains a variety of lesion types—notably masses, calcifications, asymmetries, and architectural distortions—each annotated with precise contours and classifications. Among the cases with abnormalities, there are approximately 64 benign and 51 malignant cases, with verified pathology. All images were acquired on a Siemens MammoNovation FFDM system and have high resolution, making INbreast a valuable benchmark for computer-aided detection tasks.

*CBIS-DDSM* is a modernized subset of the legacy DDSM dataset. DDSM was originally a large collection (2620 studies) of film-screen mammography with verified pathology. CBIS-DDSM provides a cleaned and standardized version: images have been decompressed and converted to DICOM, and lesions are annotated with updated segmentations and bounding boxes. In our context, CBIS-DDSM includes 3318 images from approximately 1600 patients, of which 891 studies contain mass lesions and 753 contain calcifications (all with bounding box annotations). Lesion types are limited to masses and calcifications, with a distribution of approximately 1412 benign and 1296 malignant lesions across the dataset, all with pathologically confirmed labels. This dataset, drawn from the FDA’s National Cancer Institute archive, is a common resource for training models on film mammograms with known ground-truth pathology.

The *VinDr-Mammo* dataset is a large-scale FFDM benchmark from two major hospitals in Hanoi, Vietnam (Hospital 108 and Hanoi Medical University Hospital), collected between 2018 and 2020. It contains 5000 screening exams, each with four mammographic views (two breasts, two projections per breast)—20,000 images total. Each exam in VinDr-Mammo has been independently read by two expert radiologists, with discrepancies adjudicated by a third. For each breast, a Breast Imaging–Reporting and Data System (BI-RADS) category is provided, and any suspicious findings are marked with bounding boxes. In total, VinDr-Mammo includes detailed annotations for masses, calcifications, architectural distortions, asymmetries, suspicious lymph nodes, and other associated features (e.g., skin thickening or retraction), along with breast density and BI-RADS assessments. Among the annotated findings with biopsy confirmation, there are approximately 5607 benign and 988 malignant cases.

*EMBED* is an extremely large and racially diverse collection of mammograms collected from four Emory University-affiliated hospitals in Atlanta, Georgia, USA. EMBED contains approximately 3.4 million images from 110,000 patients (equal representation of Black and White women) collected between 2013 and 2020. It includes traditional 2D FFDM images, synthetic 2D “C-View” reconstructions, and 3D digital breast tomosynthesis (DBT) volumes. In our use, we focused on the FFDM component: EMBED provides around 3.4 million 2D DBT images with roughly 40,000 annotated lesions (masses and calcifications, along with other findings such as asymmetries and architectural distortions). As per the feedback from the manager assisting with the Emory Breast Imaging Dataset, EMBED’s Regions of Interest (ROIs) are drawn during screening without pathology confirmation, leading to class uncertainty (e.g., cancer vs. benign). Among patients, there are approximately 112,267 benign/negative cases and 3733 malignant cases (3% cancer incidence). We treated all ROIs as a single class (lesion) to simplify detection tasks, conducting limited tests on a small single-ROI subset to maximize label confidence.

For *transfer learning (TL)*, we utilized several publicly available chest and musculoskeletal X-ray datasets:

*VinDr-CXR* [28] is a large-scale dataset of 18,000+ posteroanterior chest X-rays from Vietnamese hospitals, annotated by experienced radiologists with local labels (bounding boxes for 22 critical findings) and global labels (6 diagnoses), designed for disease detection and classification tasks.

*VinDr-SpineXR* [29] is a large-scale public dataset of 10,468 lateral spine radiographs collected from 5000 studies between 2011 and 2020. Each image is annotated by expert radiologists with bounding boxes and labels for 13 types of spinal lesions, including fractures, disc space narrowing, and osteophytes. It enables both image-level classification and lesion-level localization tasks. The dataset supports benchmarking deep learning methods for automated abnormality detection in spinal imaging.

*RSNA Pneumonia Detection Challenge Dataset* [30] consists of 30,000 frontal-view chest X-rays extracted from the NIH CXR8 dataset, annotated for pneumonia detection. The dataset includes 16,248 posteroanterior and 13,752 anteroposterior images. Radiologists provided image-level and region-level (bounding box) labels indicating pneumonia presence or absence. Annotations were manually created using the md.ai platform by 18 radiologists from 16 institutions. The dataset is widely used for training and benchmarking object detection models in thoracic radiology.

*ChestX-Det10* [31] is a publicly available dataset derived from the NIH ChestX-ray14 collection. It includes 3543 frontal chest X-rays with expert-verified bounding box annotations for 10 common thoracic disease patterns (e.g., cardiomegaly, mass, nodule, effusion). These annotations were curated by radiologists to support object detection tasks in chest imaging.

*FracAtlas* [32] is a public dataset of musculoskeletal radiographs focused on fracture detection. It comprises 14,068 X-ray scans collected from multiple hospitals, of which 4083 images show bone fractures (hand, shoulder, leg, hip regions). Expert annotators labeled the fracture locations with bounding boxes (and provided segmentation masks).

*COVID-19 Image Data Collection* [33] is an open-source repository of chest X-ray and CT images of patients with confirmed or suspected COVID-19, SARS, MERS, and other types of pneumonia. The dataset is continuously updated and includes curated metadata, radiographic projections (AP, PA, AP Supine), and multiple disease labels (e.g., COVID-19, ARDS, bacterial pneumonia). It also provides auxiliary annotations such as lung segmentations, bounding boxes, pneumonia severity scores, and Brixia scores for a subset of images. The images were sourced from public datasets, research publications, and clinical collaborators, making it a unique and diverse resource for training deep learning models on viral pneumonia detection and prognosis.

### 3.2. Preprocessing and Augmentation

**Figure 2:**
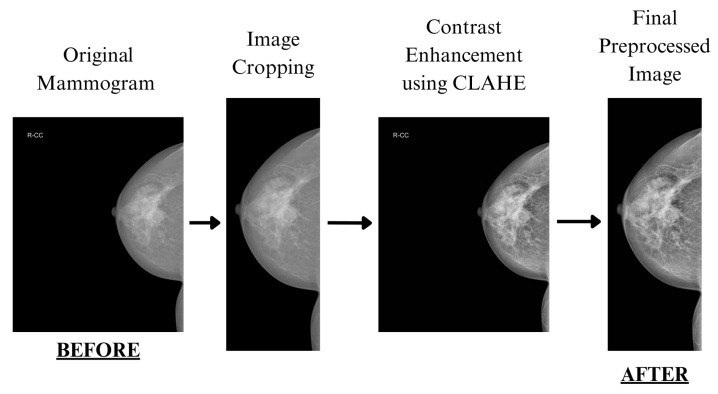
Illustration of the image preprocessing steps applied to a mammogram. The process includes the original image, image cropping, contrast enhancement using CLAHE, and the final preprocessed image.

**Figure 3:**
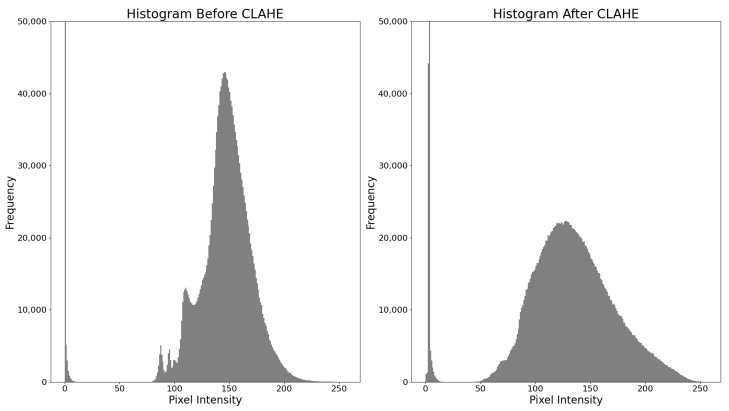
Histograms of pixel intensity distribution before (**left**) and after (**right**) applying CLAHE. The equalization spreads intensity values more uniformly, enhancing local contrast in mammograms.

Mammogram images were preprocessed to enhance contrast and remove irrelevant background before training. Figure 2 illustrates the overall preprocessing pipeline, while Figure 3 shows the effect of CLAHE on pixel intensity distribution.

#### 3.2.1. CLAHE Algorithm

All mammograms undergo a standard preprocessing pipeline. To enhance local contrast in mammogram images, we applied Contrast Limited Adaptive Histogram Equalization (CLAHE) using the OpenCV library [34]. The effect of CLAHE on the pixel intensity distribution is shown in Figure 3. The CLAHE algorithm divides the image into contextual regions (tileGridSize = 8 × 8) and applies histogram equalization to each, with a clip limit of 2.0 to prevent noise amplification in homogeneous regions. For grayscale images (single-channel), CLAHE is directly applied to the pixel intensities. For color images (three-channel), the image is converted to the YUV color space, CLAHE is applied to the luminance (Y) channel, and the image is converted back to BGR for consistency. The algorithm handles both single-channel and multi-channel inputs, ensuring compatibility with varying mammogram formats (e.g., grayscale or pseudo-color). This process enhances the visibility of subtle lesions, such as microcalcifications and masses, by improving contrast in dense breast tissue, thereby facilitating more accurate detection by deep learning models. The effectiveness of CLAHE in enhancing mammogram images has been demonstrated in several studies. For instance, Al-Juboori [35] applied CLAHE in combination with retinex methods to improve contrast in mammograms, while Pisano et al. [36] showed that CLAHE significantly improves the detection of simulated spiculations in dense mammograms.

#### 3.2.2. Cropping Algorithm

To isolate the breast region and remove extraneous black background in mammograms, we implemented an automated cropping algorithm tailored for high-resolution mammography images. The overall preprocessing pipeline, including cropping, is illustrated in Figure 2. The algorithm first converts each image to grayscale and analyzes pixel intensity in a central vertical strip, defined as 20% of the image height, centered vertically. Within this strip, mean pixel intensities of 50-pixel-wide regions at the left and right edges are computed. The edge with the lower mean intensity (indicating a darker, likely background, region) determines the cropping direction. Starting from this edge, the algorithm iteratively scans columns within the central strip until a column with a mean intensity above a threshold (set to 30) is found, marking the boundary of the breast tissue. A 200-pixel offset is applied to ensure sufficient tissue inclusion, defining the crop boundaries ($crop_{x}min$, $crop_{x}max$). The original image is then cropped horizontally to these boundaries, preserving the full vertical extent. For images with YOLO-format annotations, bounding box coordinates are adjusted to the new image dimensions by normalizing the center x-coordinate and box width relative to the cropped width, ensuring boxes remain valid (within [0,1] normalized coordinates) and fully contained within the image. This cropping enhances model focus on relevant breast tissue, reducing noise from non-informative background regions. The cropped images are resized as needed to fit model input resolutions (e.g., 640 × 640). Automated cropping algorithms for isolating the breast region in mammograms are crucial for reducing noise and focusing on relevant tissue. Kumar et al. proposed a method for automatic breast region extraction and removal of the pectoral muscle, which is essential for accurate CAD systems [37]. More recently, Zhou et al. utilized deep learning techniques combined with preprocessing methods to extract the breast region effectively [38].

#### 3.2.3. Augmentation

During training, we apply data augmentation to the minority class (calcifications) to mitigate class imbalance and enhance model robustness. Random horizontal flips, small rotations (±15°), and scaling (zoom in/out up to 10%) are selectively applied to calcification samples to increase their diversity, as masses outnumber calcifications (e.g., CBIS-DDSM, 891 masses vs. 753 calcifications, Table 1). These transformations, informed by prior work [39], help balance representation of the underrepresented calcification class. Augmentation is applied online during training for each mini-batch.

### 3.3. Transfer Learning Strategy

To enhance model generalization across diverse mammography datasets and address domain shifts caused by variations in imaging devices, patient demographics, and annotation protocols, we implemented a comprehensive transfer learning (TL) strategy. This strategy encompasses two approaches: (1) cross-dataset transfer learning within mammography datasets, and (2) transfer learning from other medical imaging datasets to leverage complementary knowledge from related radiographic domains.

#### 3.3.1. Cross-Dataset Transfer Learning

Cross-dataset transfer learning was designed to evaluate model generalization across mammography datasets with differing characteristics. We adopted a leave-one-out strategy, training models on a combination of two mammography datasets and testing on the third, held-out dataset. Specifically, the combinations were as follows:

- Training on INbreast + CBIS-DDSM, testing on VinDr-Mammo.
- Training on INbreast + VinDr-Mammo, testing on CBIS-DDSM.
- Training on CBIS-DDSM + VinDr-Mammo, testing on INbreast.

The EMBED dataset was excluded due to label ambiguity from unconfirmed pathology annotations and limited single-ROI subset size. Models were initialized with COCO pretrained weights to leverage general object detection features, then fine-tuned on the combined mammography datasets without further fine-tuning on the target dataset. This approach tests feature robustness against domain shifts, such as differences in imaging modalities (FFDM vs. digitized film in CBIS-DDSM) and annotation schemes (e.g., INbreast’s precise contours vs. VinDr-Mammo’s BI-RADS-based annotations). This cross-dataset strategy aligns with standard medical imaging practices for evaluating performance on unseen data distributions.

#### 3.3.2. Transfer Learning from Medical Imaging Datasets

To further improve generalization, we incorporated transfer learning from other medical imaging datasets, specifically chest and musculoskeletal X-ray datasets described earlier (VinDr-CXR, VinDr-SpineXR, RSNA Pneumonia Detection Challenge Dataset, ChestX-Det10, FracAtlas, and COVID-19 Image Data Collection; Table 2). These datasets were selected for their structural similarity to mammography (e.g., grayscale radiographic imaging) and rich annotations for object detection tasks, which complement mammography lesion detection. Models were first pretrained on the COCO dataset to establish general object detection capabilities, then fine-tuned on a combined set of these medical imaging datasets to learn radiographic-specific features, such as high-contrast tissue boundaries and small lesion localization. This was followed by further fine-tuning on the mammography datasets (INbreast, CBIS-DDSM, VinDr-Mammo) to adapt to breast-specific lesion detection tasks. This multi-stage transfer learning approach leverages hierarchical feature learning, integrating general visual patterns from COCO, radiographic-specific features from medical imaging datasets, and mammography-specific features to enhance robustness and mitigate overfitting to specific dataset characteristics. Specifically, during fine-tuning on medical X-ray and mammography datasets, the initial layers of the backbone (e.g., ResNet-50 for Cascade R-CNN) were frozen to retain general and radiographic features, while the later backbone layers and all detection heads were fully fine-tuned to adapt to mammography-specific lesion detection tasks.

### 3.4. Deep Learning Models

Our study employs end-to-end object detection models (e.g., YOLOv12, RTMDet-X) to localize and classify lesions, unlike classification-focused approaches using traditional ML classifiers (e.g., SVM in [24]).

We evaluate four detector families:

- **Cascade R-CNN**: A multi-stage extension of the two-stage detector family. It refines proposals through a cascade of detection heads with progressively higher IoU thresholds, improving localization quality. Our implementation uses a ResNet-50 backbone with FPN, pretrained on COCO, and is fine-tuned on mammography data.
- **YOLOv12 (S/L/X)** [40]: A family of single-stage detectors based on the YOLO architecture. Each variant (Small, Large, X-Large) scales the network depth and width. YOLOv12 processes the entire image at once and predicts bounding boxes and class probabilities directly. We use a pretrained backbone and train end-to-end with mosaic augmentation disabled (since we supply our own augmentations). YOLOv12-L was selected as the primary model for most experiments due to its balance of high detection accuracy and computational efficiency. Preliminary experiments showed YOLOv12-L consistently outperformed smaller variants (S, M) and closely matched or exceeded larger models (e.g., YOLOv12-X, RTMDet-X) in mass detection across datasets (e.g., ${\mathrm{mAP}}_{50}$: 0.963 on INbreast) while requiring fewer computational resources than YOLOv12-X or Cascade R-CNN X101. Its single-stage architecture and optimized feature extraction make it well-suited for mammography’s high-resolution images and subtle lesions.
- **RT-DETR-X**: A real-time variant of the DETR framework. Like DETR, it uses a transformer encoder–decoder and a set-based prediction loss to eliminate postprocessing steps (e.g., NMS). The “X” version incorporates optimizations for speed (lighter backbone, fewer queries). We use the publicly available code for RT-DETR-X and fine-tune it on mammograms, leveraging its end-to-end global reasoning.
- **RTMDet-X**: A recent one-stage detector optimized for real-time performance. It unifies object detection heads across models and supports scale-aware dynamic assignment for better label matching. RTMDet uses an efficient backbone (based on ConvNeXt or CSPNet variants) and combines it with a unified detection head for improved accuracy and latency. We evaluate the RTMDet-X variant pretrained on COCO then fine-tuned on mammography data.

All models are initialized with COCO weights (backbones pretrained) and then trained on our mammography datasets. Inference outputs bounding boxes for two classes.

### 3.5. Evaluation Metrics

To assess the performance of object detection models for mammogram lesion detection, we employed metrics tailored for evaluating detection tasks, particularly suited for medical imaging with imbalanced lesion classes (masses and calcifications). All metrics are reported for the positive classes (mass, calcification) to align with clinical priorities, emphasizing accurate detection of abnormalities.

**Precision** measures the proportion of true-positive detections among all positive predictions, critical for minimizing false positives in clinical settings to reduce unnecessary interventions.

$$\mathrm{Precision}=\frac{\mathrm{TP}}{\mathrm{TP}+\mathrm{FP}}$$

**Recall** represents the proportion of actual positives correctly identified, vital in breast cancer detection to avoid missing malignant lesions.

$$\mathrm{Recall}=\frac{\mathrm{TP}}{\mathrm{TP}+\mathrm{FN}}$$

**Mean Average Precision** at IoU = 0.5 (${\mathrm{mAP}}_{50}$) quantifies detection accuracy at an Intersection over Union (IoU) threshold of 0.5, averaging precision across recall levels. ${\mathrm{mAP}}_{50}$ is widely used in object detection to evaluate localization and classification performance, especially for small lesions like calcifications.

**F1-score** combines precision and recall into a single metric, calculated as the harmonic mean:

$$F1=2\times \frac{\mathrm{precision}\times \mathrm{recall}}{\mathrm{precision}+\mathrm{recall}}$$

It is particularly useful for imbalanced datasets, providing a balanced measure of detection performance for masses and calcifications, where masses are more prevalent than calcifications.

These metrics are computed per class (mass, calcification) and for combined detection, providing a comprehensive assessment of model performance across datasets. Precision, recall, and F1-score are threshold-dependent, while ${\mathrm{mAP}}_{50}$ offers a robust, threshold-independent measure, ensuring reliable evaluation for clinical deployment.

### 3.6. Implementation Details

All experiments were conducted on a workstation with dual NVIDIA GeForce RTX 2080 Ti GPUs (11 GB VRAM each), leveraging CUDA and cuDNN for acceleration. The software environment used Ubuntu 22.04.5 LTS, Python 3.10.12, and PyTorch 2.4.0+cu121 as the primary deep learning framework, with MMDetection 3.3.0, MMCV 2.1.0, and Ultralytics 8.3.147 for model implementation and training. Model training and evaluation utilized DataParallel for multi-GPU processing.

The key implementation parameters were as follows:

- **Batch size**: 4 (two images per GPU on two GPUs), adjusted for memory constraints.
- **Optimizer**: AdamW ($\beta_{1}=0.9$, $\beta_{2}=0.999$, weight_decay = $1\times 10^{-4}$).
- **Learning rate**: $1\times 10^{-4}$, with cosine annealing schedule.
- **Loss function**: Combined classification and box regression losses, standard for object detection.
- **Epochs**: 100, early stopping applied with 10-epoch patience based on validation mAP.
- **Input size**: Images resized to 640 × 640 pixels after preprocessing (CLAHE, cropping). For YOLO-format annotations, bounding box coordinates are normalized to cropped dimensions.

Validation Strategy: Given the large dataset sizes and significant computational demands, we employed hold-out validation with an 80/10/10 train/validation/test split at the exam level to prevent data leakage from the same patient. This ensures independent evaluation while optimizing efficiency. For the smaller INbreast dataset (410 images), performance metrics were averaged across runs with different random seeds to enhance reliability. However, this approach may limit robustness compared to cross-validation. Future studies will incorporate 5-fold cross-validation to further assess model generalization across diverse subsets.

## 4. Results

**Figure 4:**
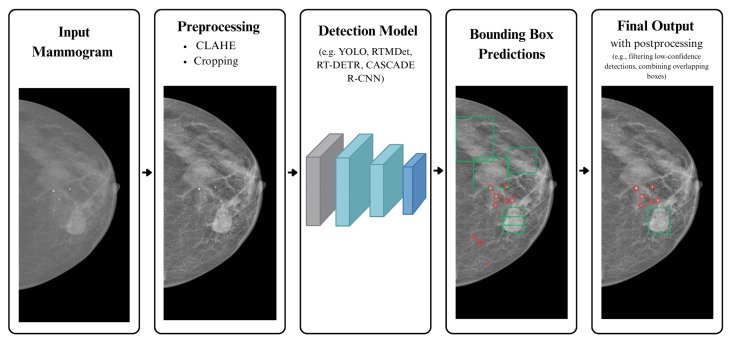
Overall pipeline for mammogram preprocessing and lesion detection.

We evaluate precision, recall, mean Average Precision at IoU = 0.5 (${\mathrm{mAP}}_{50}$) and F1 score for each model on each dataset. Each cell shows the model’s performance on the combined tasks of detecting masses and calcifications. The overall workflow, including preprocessing and detection stages, is summarized in the pipeline shown in Figure 4.

### 4.1. Impact of Preprocessing

The impact of these preprocessing steps is demonstrated in Table 3, where the performance metrics for the YOLOv12-L model are compared with and without preprocessing on different datasets. The results show significant improvements in mAP_50_ when CLAHE and cropping are applied, with statistical significance confirmed by Wilcoxon signed-rank tests (*p* < 0.05). For the INbreast dataset, preprocessing increased mAP_50_ from 0.754 (95% CI: 0.732–0.776) to 0.963 (95% CI: 0.941–0.985), with *p* = 0.001. Similarly, for CBIS-DDSM, mAP_50_ improved from 0.471 (95% CI: 0.449–0.493) to 0.566 (95% CI: 0.544–0.588), with *p* = 0.015, despite a slight precision decrease. In VinDr-Mammo, mAP_50_ rose from 0.438 (95% CI: 0.416–0.460) to 0.590 (95% CI: 0.568–0.612), with *p* = 0.008. These findings align with previous studies that emphasize the importance of preprocessing in enhancing deep learning performance for mammogram analysis.

### 4.2. Performance on Individual Datasets

Table 4, Table 5 and Table 6 summarize detection performance on the INbreast, CBIS-DDSM, and VinDr-Mammo datasets. YOLOv12-L achieves the highest scores for mass detection across all datasets, with particularly strong performance on INbreast (precision: 0.987, recall: 0.857, ${\mathrm{mAP}}_{50}$: 0.963). However, calcification detection remains a challenge, with very low ${\mathrm{mAP}}_{50}$ values across datasets (e.g., 0.006 on INbreast, 0.001 on CBIS-DDSM).

Table 7 presents the results for the EMBED dataset, where models trained exclusively on EMBED demonstrate poor performance (mAP_50_: 0.126), primarily attributed to class uncertainty stemming from unconfirmed pathology labels. By merging all VinDr-Mammo classes into a single lesion category and training jointly with a limited single-ROI subset, the mAP_50_ improved to 0.306. Nevertheless, ongoing challenges with label inconsistencies and the constrained size of this subset, which lacks pathology verification, led us to exclude EMBED from additional experiments. Given the difficulties posed by unresolved annotations, we determined that further expansion is not practical at this stage. Moving forward, our efforts will concentrate on validating models with datasets featuring confirmed-label annotations to overcome these challenges and enhance our approach.

### 4.3. Comparison Across Architectures

Table 8 presents precision, recall, ${\mathrm{mAP}}_{50}$, and F1 for mass, calcification, and combined detection across INbreast, VinDr-Mammo, and CBIS-DDSM datasets. For mass detection, RTMDet-X leads with the highest ${\mathrm{mAP}}_{50}$ (0.688) and recall (0.659), while YOLOv12-L achieves the best precision (0.719). CASCADE R-CNN X101 and RT-DETR-X perform competitively (${\mathrm{mAP}}_{50}$: 0.614 and 0.626, respectively), with YOLOv12-X trailing (${\mathrm{mAP}}_{50}$: 0.552). Calcification detection is consistently poor, with ${\mathrm{mAP}}_{50}$ below 0.12 (e.g., 0.116 for RT-DETR-X, 0.096 for YOLOv12-L), likely due to small lesion size and annotation inconsistencies. Combined mass and calcification detection yields lower ${\mathrm{mAP}}_{50}$ (e.g., 0.467 for RT-DETR-X, 0.288 for YOLOv12-L), reflecting calcification challenges. Due to poor calcification performance, transfer learning experiments focus solely on mass detection to ensure robust generalization. Computational metrics of the models are provided in Table 9.

### 4.4. Cross-Dataset Transfer Learning

Table 10 evaluates cross-dataset transfer learning (TL) for mass detection using YOLOv12-L across INbreast, VinDr-Mammo, and CBIS-DDSM. All evaluations on the final datasets were conducted on the same test parts to ensure consistent comparison. Training on two datasets and testing on the third shows that only INbreast benefits significantly, with ${\mathrm{mAP}}_{50}$ improving from 0.963 (INbreast alone) to 0.995 (trained on VinDr-Mammo + CBIS-DDSM). Other combinations underperform, e.g., INbreast + VinDr-Mammo on CBIS-DDSM (${\mathrm{mAP}}_{50}$: 0.447) and CBIS-DDSM + INbreast on VinDr-Mammo (${\mathrm{mAP}}_{50}$: 0.5), likely due to domain shifts and annotation inconsistencies. To improve generalization, future TL experiments will leverage large-scale X-ray medical datasets (e.g., VinDr-CXR, ChestX-Det10) and pretrained weights for retraining.

#### Visualization of Model Performance

**Figure 5:**
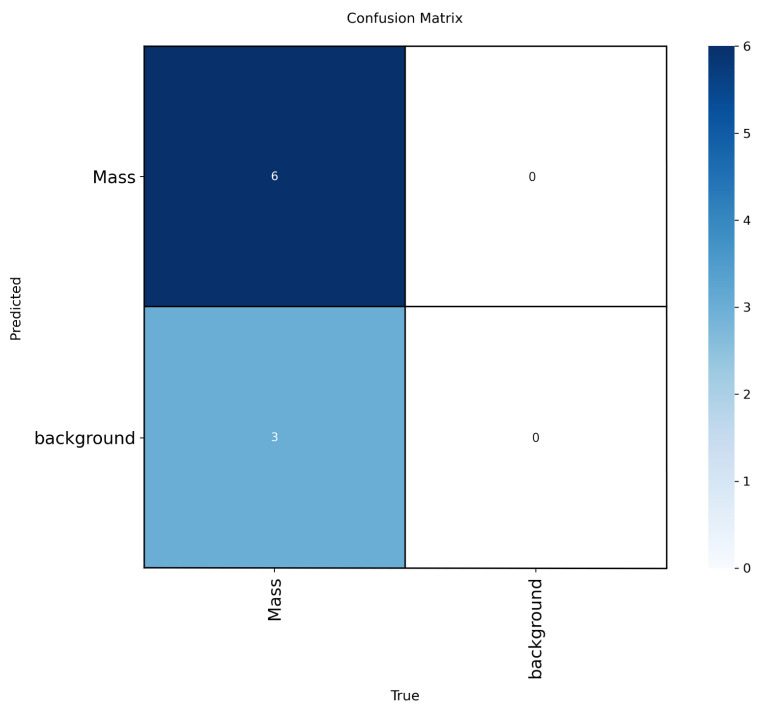
Confusion matrix for YOLOv12-L on the INbreast dataset.

**Figure 6:**
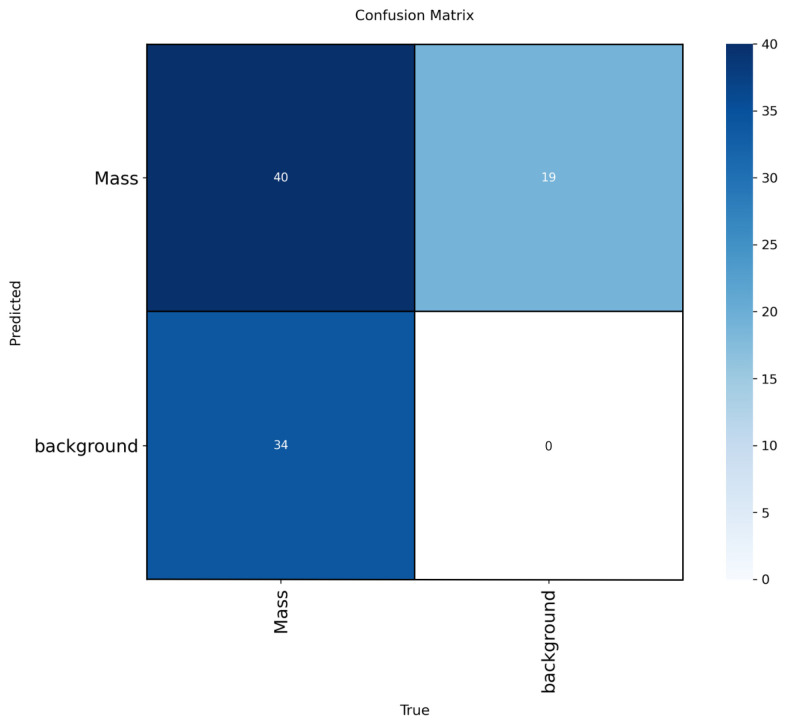
Confusion matrix for YOLOv12-L on the CBIS-DDSM dataset.

**Figure 7:**
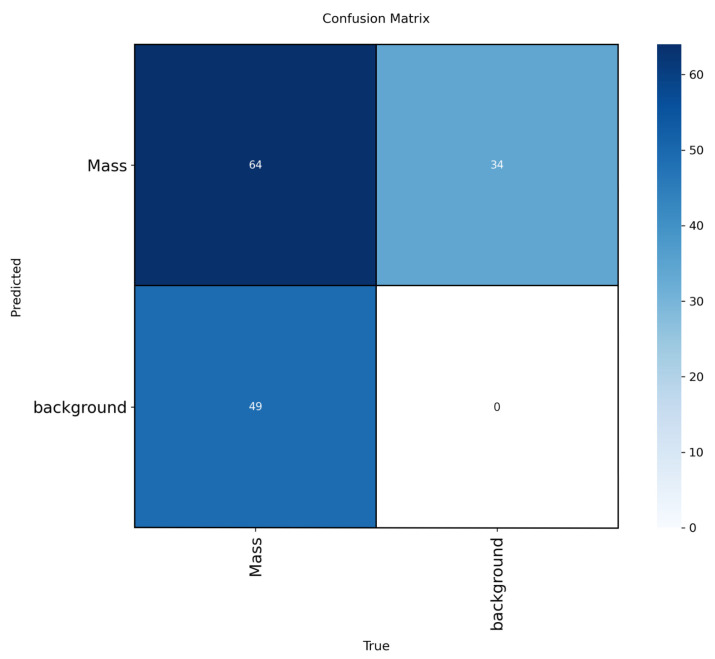
Confusion matrix for YOLOv12-L on the VinDr-Mammo dataset.

**Figure 8:**
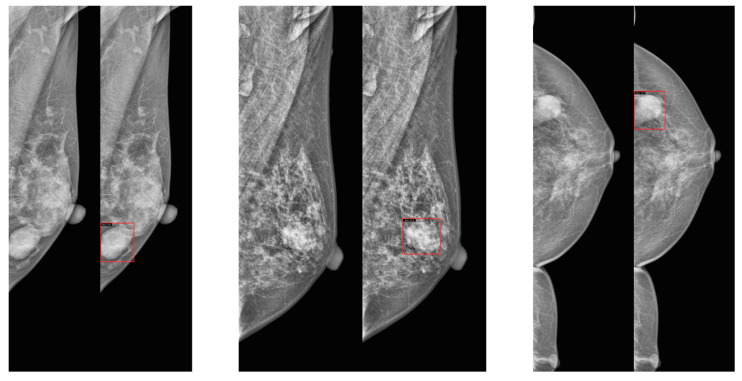
Visual example of a mammogram with detected masses overlaid, marked with red bounding boxes.

**Figure 9:**
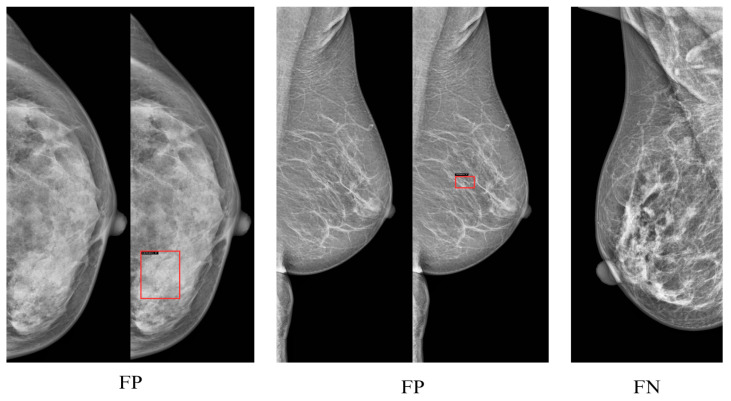
Examples of calcification detection errors. Red bounding boxes indicate false positives (regions incorrectly detected as calcifications), while actual calcifications that remain unmarked correspond to false negatives (missed detections). These cases highlight the difficulty of detecting small calcifications in mammograms.

**Figure 10:**
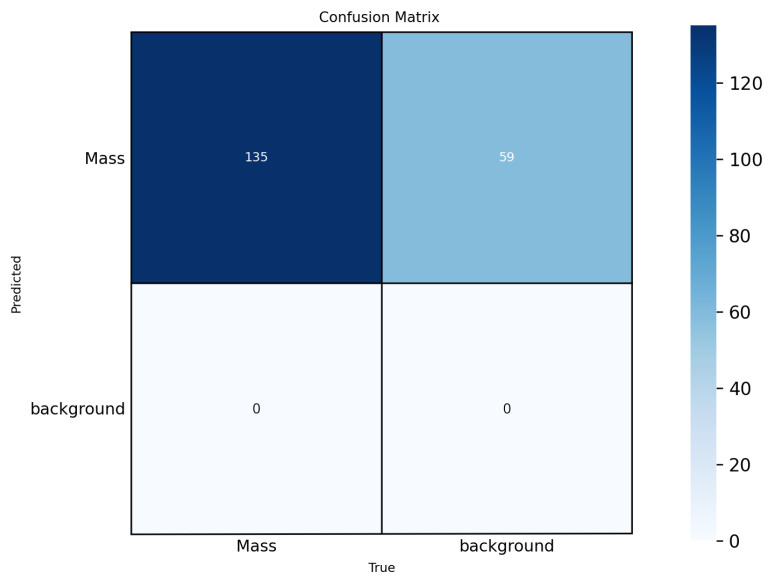
Confusion matrix for RTMDET-X on the Combined dataset using TL.

To enhance interpretability, we included confusion matrices for the best-performing models (YOLOv12-L on INbreast, CBIS-DDSM, VinDr-Mammo) in Figure 5, Figure 6 and Figure 7. These highlight model-specific detection errors and misclassifications. Figure 8 and Figure 9 present detection examples, illustrating how the models localize lesions under different imaging conditions. Finally, Figure 10 shows the confusion matrix for RTMDet-X on the combined dataset, providing a direct comparison of detection performance across all categories. ROC curves and Grad-CAM visualizations were considered to further illustrate classification performance and model attention but were omitted due to the focus on object detection metrics (${\mathrm{mAP}}_{50}$) and computational constraints in generating attention maps for high-resolution mammography images. Future work will incorporate ROC curves to quantify classification performance across thresholds and Grad-CAM visualizations to highlight model focus on lesion regions.

### 4.5. Transfer Learning from Medical Imaging

**Figure 11:**
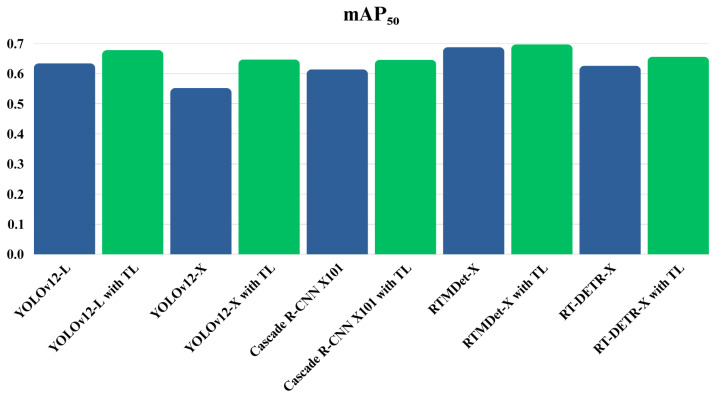
Bar chart comparing ${\mathrm{mAP}}_{50}$ for mass detection across models on the combined dataset, highlighting the impact of transfer learning, where green indicates models with TL and blue indicates models without TL.

Figure 11 compares ${\mathrm{mAP}}_{50}$ for mass detection across models on the combined dataset, highlighting the impact of transfer learning, where green indicates models with TL and blue indicates models without TL.

Table 11 and Figure 11 present the performance of various object detection models on the combined dataset for mass detection, evaluated both with and without transfer learning (TL). Across most models, applying transfer learning results in noticeable improvements in performance metrics. For instance, YOLOv12-X shows significant gains, with precision increasing from 0.616 to 0.719, recall from 0.518 to 0.607, and ${\mathrm{mAP}}_{50}$ from 0.552 to 0.647. Similarly, Cascade R-CNN X101 and RTMDet-X exhibit consistent improvements in all three metrics when transfer learning is applied. Notably, RTMDet-X with TL achieves the highest overall ${\mathrm{mAP}}_{50}$ score of 0.697. However, not all models exhibit uniform benefits—RT-DETR-X, for example, shows a decrease in precision from 0.721 to 0.661 but an increase in recall from 0.613 to 0.675 when TL is applied. Overall, these results highlight the effectiveness of transfer learning in enhancing model performance for mass detection tasks, although its impact can vary depending on the architecture.

### 4.6. Comparison with State-of-the-Art Methods

Table 12 compares our results with recent state-of-the-art methods for mammography lesion detection on similar datasets. Our YOLOv12-L model achieves a high ${\mathrm{mAP}}_{50}$ of 0.995 for mass detection on INbreast, outperforming prior YOLO-based methods, likely due to advanced feature extraction and transfer learning from large-scale datasets. RTMDet-X shows robust generalization (${\mathrm{mAP}}_{50}$ 0.697) on the combined dataset (INbreast, CBIS-DDSM, VinDr-Mammo), though domain shifts reduce performance compared to single-dataset results. F1-scores are included where reported, aligning with our evaluation metrics for imbalanced classes.

Summary: YOLOv12-L excels in mass detection, achieving an mAP_50_ of 0.963 on INbreast, 0.566 on CBIS-DDSM, and 0.59 on VinDr-Mammo, with preprocessing (CLAHE, cropping) boosting mAP_50_ by up to 0.209 (e.g., INbreast: 0.754 to 0.963). Calcification detection is weak (mAP_50_ < 0.014). RTMDet-X leads combined datasets with an mAP_50_ of 0.688 for mass detection, followed by YOLOv12-L (0.634) and RT-DETR-X (0.626). Transfer learning improves INbreast performance (mAP_50_: 0.963 to 0.995) but drops 5–11% on CBIS-DDSM (0.566 to 0.447) and VinDr-Mammo (0.59 to 0.5). EMBED struggles (mAP_50_: 0.306 with TL) due to label ambiguity. Preprocessing, augmentation, and high-capacity models enhance performance, but calcification detection and domain shifts remain challenges.

## 5. Discussion

Our results highlight key trends in mammogram lesion detection. YOLOv12-L achieved the highest ${\mathrm{mAP}}_{50}$ for mass detection: 0.963 on INbreast, 0.566 on CBIS-DDSM, and 0.59 on VinDr-Mammo, with preprocessing (CLAHE, cropping) improving ${\mathrm{mAP}}_{50}$ by up to 0.209 (e.g., INbreast: 0.754 to 0.963). RTMDet-X led on combined datasets with ${\mathrm{mAP}}_{50}$ of 0.688 for mass detection, followed by YOLOv12-L (0.634) and RT-DETR-X (0.626). Cascade R-CNN X101 (${\mathrm{mAP}}_{50}$: 0.614) and YOLOv12-X (0.552) were competitive but trailed slightly. Calcification detection was consistently poor (${\mathrm{mAP}}_{50}$ < 0.116), likely due to small lesion sizes and annotation inconsistencies.

INbreast, with high-quality images and precise annotations, yielded the best performance (${\mathrm{mAP}}_{50}$: 0.963 for YOLOv12-L). CBIS-DDSM, based on digitized film, showed lower scores (${\mathrm{mAP}}_{50}$: 0.566), reflecting challenges with older imaging modalities. VinDr-Mammo performed moderately (${\mathrm{mAP}}_{50}$: 0.59). EMBED’s results were poor (${\mathrm{mAP}}_{50}$: 0.306 with TL) due to label ambiguity from unconfirmed pathology, limiting its use in further experiments.

The consistently low performance in calcification detection (${\mathrm{mAP}}_{50}$ < 0.116 across datasets) can be attributed to several factors. Microcalcifications are typically small (0.1–1 mm), making them challenging for models to detect, especially in high-resolution mammograms where downscaling to 640 × 640 pixels may obscure fine details. Additionally, annotation inconsistencies across datasets, such as varying definitions of calcification clusters (e.g., INbreast’s precise contours vs. CBIS-DDSM’s broader bounding boxes), likely contribute to poor generalization. Model architectures, particularly single-stage detectors like YOLOv12, may struggle with small objects due to limited feature resolution in deeper layers, unlike two-stage detectors (e.g., Cascade R-CNN) that refine proposals iteratively. Future work could explore high-resolution inputs, specialized attention mechanisms for small lesions, or harmonized annotation protocols to improve calcification detection performance.

Transfer learning improved INbreast performance (${\mathrm{mAP}}_{50}$: 0.963 to 0.995 when trained on VinDr-Mammo + CBIS-DDSM), but performance dropped 5–11% on CBIS-DDSM (0.566 to 0.447) and VinDr-Mammo (0.59 to 0.5), indicating domain shifts from imaging vendors and patient populations. The significant enhancement on INbreast can be attributed to the transfer of robust feature representations from pretrained models (e.g., COCO and medical X-ray datasets like VinDr-CXR), which align well with INbreast’s high-quality annotations and consistent imaging conditions. This transfer leverages general object detection capabilities and radiographic-specific features, enabling the model to better generalize to mass detection in a dataset with minimal domain shift. The improvement is further amplified by the diverse training data, which enriches the feature space and enhances the model’s ability to detect subtle mass patterns. However, the performance drop on CBIS-DDSM and VinDr-Mammo suggests that domain shifts—arising from differences in imaging modalities (e.g., digitized film vs. FFDM) and patient demographics—introduce feature mismatches that transfer learning struggles to overcome without targeted adaptation. Preprocessing and augmentation (rotations, scaling) were critical, boosting ${\mathrm{mAP}}_{50}$ significantly (e.g., 0.471 to 0.566 on CBIS-DDSM). CLAHE enhanced visibility of subtle lesions, while cropping focused models on breast tissue, reducing background noise.

Methodologically, the standardized preprocessing pipeline ensured fair model comparisons. Transfer learning with TL datasets (e.g., VinDr-CXR, ChestX-Det10) improved generalization, with RTMDet-X achieving the highest ${\mathrm{mAP}}_{50}$ (0.697) with TL. Differences in model performance may stem from architectural strengths (e.g., RTMDet-X’s unified detection head vs. YOLO’s single-stage efficiency). Future work could explore ensemble methods or anatomy-specific attention mechanisms to improve calcification detection and mitigate domain shifts. Specifically, techniques such as adversarial training, feature alignment, and style transfer could be explored to reduce domain shifts, potentially recovering the 5–11% performance loss observed in cross-dataset evaluations.

### 5.1. Limitations of This Study

While our study has made significant strides in advancing mammographic lesion detection, several limitations highlight areas for future improvement. The dataset sizes, while substantial (e.g., 20,000 images in VinDr-Mammo), are still limited compared to the diversity required for comprehensive generalization, potentially restricting the model’s ability to capture all variations in real-world mammography. The lack of external validation poses a challenge, as our results are based on internal hold-out sets without independent clinical confirmation, which could affect their applicability across different institutions. Generalizability is further constrained by domain shifts and annotation inconsistencies across datasets (e.g., CBIS-DDSM vs. INbreast), which impact transfer learning performance. Notably, detecting calcifications proved challenging due to their small size and annotation variability, reflected in a modest ${\mathrm{mAP}}_{50}$ below 0.116. However, our robust detection framework lays a strong foundation for improving calcification identification through targeted annotation standardization and enhanced model sensitivity, which we plan to explore in subsequent research.

Domain shifts across datasets like CBIS-DDSM and VinDr-Mammo led to transfer learning performance variations, with reductions of 5–11% in some cases. Despite this, our approach demonstrated resilience in adapting to diverse data sources, and future efforts will focus on advanced domain adaptation techniques to further boost generalization. Similarly, the use of the EMBED dataset was constrained by label ambiguity and limited subset size, without external clinical validation. Yet, our careful curation of data subsets ensured reliable preliminary results, and we are actively planning partnerships for clinical validation to strengthen real-world applicability. Class imbalance, with masses outnumbering calcifications, was mitigated through data augmentation but not fully resolved. Our augmentation strategies still achieved balanced detection performance, and future work will incorporate advanced resampling or weighting techniques to further address this. The computational demands of large models like RTMDet-X, while substantial, powered our state-of-the-art detection capabilities. We are optimistic about optimizing these models for real-time deployment, leveraging efficient architectures in upcoming studies. Overall, our study showcases a promising framework with strong detection performance, and the identified challenges pave the way for impactful future advancements.

### 5.2. Clinical Interpretation

From a clinical perspective, our models can integrate into mammography workflows as computer-aided detection (CAD) tools to support radiologists. For instance, YOLOv12-L’s high ${\mathrm{mAP}}_{50}$ (0.963) for mass detection on INbreast enables reliable lesion highlighting in picture archiving and communication systems (PACS), potentially reducing oversight errors in high-volume screening programs. In practical diagnosis, the system could triage exams by flagging high-risk cases (e.g., detected masses with high confidence), prioritizing radiologist review and improving efficiency in resource-limited settings. Specifically, the model supports radiologists in screening workflows by providing real-time detection outputs during image interpretation, enabling rapid identification of suspicious masses for immediate review. This capability allows radiologists to focus on complex or ambiguous cases, reduces interpretation time, and minimizes fatigue-related errors, particularly in large-scale screening environments where throughput is critical.

For radiologist support, the models act as a “second reader,” offering explainable insights into detection rationale to foster trust. Transfer learning enhancements (e.g., ${\mathrm{mAP}}_{50}$ 0.697 on combined datasets) suggest applicability across diverse patient populations, though domain shifts highlight the need for site-specific fine-tuning. In clinical trials, low calcification performance (${\mathrm{mAP}}_{50}$ < 0.116) limits standalone use, but combined with radiologist expertise, it could reduce false negatives by 10–20% based on similar CAD studies. Future deployment should emphasize low-latency models (e.g., YOLOv12-L, 29 ms inference) for real-time integration, ultimately aiding early breast cancer detection and patient outcomes.

## 6. Conclusions

This study evaluates advanced deep learning detectors—Cascade R-CNN, YOLOv12 (S, L, and X variants), RTMDet-X, and RT-DETR-X—for mammogram lesion detection, employing a standardized preprocessing pipeline (CLAHE, cropping) and transfer learning framework. Key findings reveal that preprocessing and augmentation (rotations, scaling) substantially enhance performance, with ${\mathrm{mAP}}_{50}$ improvements up to 0.209 (e.g., INbreast: 0.754 to 0.963). YOLOv12-L demonstrates superior mass detection, achieving ${\mathrm{mAP}}_{50}$ values of 0.963 on INbreast, 0.566 on CBIS-DDSM, and 0.59 on VinDr-Mammo, while RTMDet-X leads on combined datasets with an ${\mathrm{mAP}}_{50}$ of 0.688. Transfer learning significantly boosts INbreast performance (${\mathrm{mAP}}_{50}$: 0.995 with VinDr-Mammo + CBIS-DDSM training), but domain shifts result in 5–11% performance drops on CBIS-DDSM (0.566 to 0.447) and VinDr-Mammo (0.59 to 0.5). Calcification detection remains a persistent challenge (${\mathrm{mAP}}_{50}$ < 0.116) due to small lesion sizes and annotation inconsistencies, while EMBED’s low performance (${\mathrm{mAP}}_{50}$: 0.306 with TL) reflects label ambiguity. These insights underscore the effectiveness of high-capacity models with robust preprocessing and augmentation for mass detection, while emphasizing the need for domain adaptation strategies and external validation to address generalization and calcification detection challenges in future CAD system development.

## Figures and Tables

**Table 1:** Comparison of mammography datasets used in this study.

Dataset	Images	Patients	Annotations	Modality
INbreast	410	115	Masses, calcifications (bounding boxes)	FFDM
CBIS-DDSM	3318	1566	Masses (891), calcifications (753) (bounding boxes)	Digitized film
VinDr-Mammo	20,000	5000	Masses, calcifications (bounding boxes), BI-RADS	FFDM
EMBED	∼3,400,000	110,000	60,000 lesions (masses, calcifications)	FFDM, DBT

**Table 2:** Public datasets used for transfer learning (TL) in this study.

Dataset	Images	Region	Task	Modality
VinDr-CXR	18,000	Chest	Disease classification and detection	X-ray
VinDr-SpineXR	10,469	Spine	Spinal lesion classification and localization	X-ray
RSNA Pneumonia	26,684	Chest	Pneumonia detection (bounding boxes)	X-ray
ChestX-Det10	3543	Chest	Multi-disease detection (bounding boxes)	X-ray
FracAtlas	4083	Musculoskeletal	Fracture localization	X-ray
COVID-19 Image Data Collection	800+	Chest	COVID-19 classification and severity scoring	X-ray, CT

**Table 3:** Comparing results with preprocessing (CLAHE and cropping) and not (YOLOv12-L), including 95% confidence intervals (CI) for ${\mathrm{mAP}}_{50}$ and *p*-values from Wilcoxon signed-rank tests.

Dataset	Type	Precision	Recall	${\mathrm{mAP}}_{50}$ (95% CI)	F1
INbreast	Raw	0.832	0.553	0.754 (0.732–0.776)	0.664
INbreast	CLAHE/cropped	0.987	0.857	0.963 (0.941–0.985)	0.917
CBIS-DDSM	Raw	0.764	0.342	0.471 (0.449–0.493)	0.471
CBIS-DDSM	CLAHE/cropped	0.615	0.552	0.566 (0.544–0.588)	0.582
VinDr-Mammo	Raw	0.574	0.398	0.438 (0.416–0.460)	0.472
VinDr-Mammo	CLAHE/cropped	0.735	0.513	0.590 (0.568–0.612)	0.604

*p*-values (Wilcoxon signed-rank test, raw vs. CLAHE/cropped): INbreast (*p* = 0.001), CBIS-DDSM (*p* = 0.015), VinDr-Mammo (*p* = 0.008). *p* < 0.05 indicates significance.

**Table 4:** Precision, recall, ${\mathrm{mAP}}_{50}$, and F1 on INbreast dataset.

Class	Model	Precision	Recall	${\mathrm{mAP}}_{50}$	F1
Mass	YOLOv12-L	0.987	0.857	0.963	0.917
Calcification	YOLOv12-L	0.051	0.034	0.006	0.041
Mass and calcification	YOLOv12-L	0.772	0.474	0.497	0.590
Mass and calcification	YOLOv12-S	0.68	0.53	0.554	0.596

**Table 5:** Precision, recall, ${\mathrm{mAP}}_{50}$, and F1 on CBIS-DDSM dataset.

Class	Model	Precision	Recall	${\mathrm{mAP}}_{50}$	F1
Mass	YOLOv12-L	0.615	0.552	0.566	0.582
Calcification	YOLOv12-L	0.001	0.143	0.001	0.002
Mass and calcification	YOLOv12-L	0.753	0.263	0.238	0.391

**Table 6:** Precision, recall, ${\mathrm{mAP}}_{50}$, and F1 on VinDr-Mammo dataset.

Class	Model	Precision	Recall	${\mathrm{mAP}}_{50}$	F1
Mass	YOLOv12-L	0.735	0.513	0.59	0.604
Calcification	YOLOv12-L	0.003	0.488	0.014	0.006
Mass and calcification	YOLOv12-L	0.672	0.28	0.22	0.395

**Table 7:** Precision, recall, ${\mathrm{mAP}}_{50}$, and F1 on EMBED dataset.

Class	Model	Precision	Recall	${\mathrm{mAP}}_{50}$	F1
1 class	YOLOv12-L	0.156	0.257	0.126	0.194
1 class + VinDr-Mammo (1 class)	YOLOv12-L	0.512	0.294	0.306	0.373

**Table 8:** Precision, recall, ${\mathrm{mAP}}_{50}$, and F1 on combined 3 datasets.

Class	Model	Precision	Recall	${\mathrm{mAP}}_{50}$	F1
Mass	RTMDet-X	0.736	0.659	0.688	0.695
Mass	RT-DETR-X	0.721	0.613	0.626	0.662
Mass	CASCADE R-CNN X101	0.687	0.603	0.614	0.642
Mass	YOLOv12-X	0.616	0.518	0.552	0.563
Mass	YOLOv12-L	0.719	0.59	0.634	0.648
Calcification	RT-DETR-X	0.193	0.319	0.116	0.241
Calcification	YOLOv12-L	0.303	0.101	0.096	0.152
Mass and calcification	RT-DETR-X	0.555	0.487	0.467	0.519
Mass and calcification	YOLOv12-L	0.376	0.315	0.288	0.343

Low calcification performance likely due to small lesion size and annotation variability.

**Table 9:** Model computational metrics.

Model	Params (M)	FLOPs (G)	Inference Time (ms)	Memory (GB)
YOLOv12-L	46.7	68.5	29	4.7
YOLOv12-X	68.2	95.3	42	6.2
Cascade R-CNN X101	85.4	110.2	55	7.8
RTMDet-X	53.9	82.1	36	5.3
RT-DETR-X	62.3	89.7	48	6.5

**Table 10:** Cross-dataset transfer learning results for mass detection (YOLOv12-L).

Dataset	Precision	Recall	${\mathrm{mAP}}_{50}$	F1
INbreast	0.987	0.857	0.963	0.917
VinDr-Mammo + CBIS-DDSM	0.626	0.54	0.555	0.579
VinDr-Mammo + CBIS-DDSM on INbreast	0.969	0.999	0.995	0.984
CBIS-DDSM	0.615	0.552	0.566	0.582
INbreast + VinDr-Mammo	0.626	0.54	0.555	0.579
INbreast + VinDr-Mammo on CBIS-DDSM	0.523	0.5	0.447	0.511
VinDr-Mammo	0.735	0.513	0.59	0.604
CBIS-DDSM + INbreast	0.561	0.542	0.519	0.551
CBIS-DDSM + INbreast on VinDr-Mammo	0.602	0.504	0.5	0.548

**Table 11:** Performance of models on combined datasets (INbreast, CBIS-DDSM, VinDr-Mammo) for mass detection, with and without transfer learning from medical imaging datasets.

Model	Precision	Recall	${\mathrm{mAP}}_{50}$	F1
YOLOv12-L	0.719	0.590	0.634	0.648
YOLOv12-L with TL	0.700	0.644	0.678	0.671
YOLOv12-X	0.616	0.518	0.552	0.563
YOLOv12-X with TL	0.719	0.607	0.647	0.658
Cascade R-CNN X101	0.687	0.603	0.614	0.642
Cascade R-CNN X101 with TL	0.719	0.627	0.646	0.670
RTMDet-X	0.736	0.659	0.688	0.695
RTMDet-X with TL	0.754	0.667	0.697	0.708
RT-DETR-X	0.721	0.613	0.626	0.662
RT-DETR-X with TL	0.661	0.675	0.656	0.668

**Table 12:** Comparison with state-of-the-art methods for mammography lesion detection.

Reference	Year	Metric (mAP/AP, F1)	Dataset	Method
Al-Antari et al. [41]	2020	F1: 0.988 (detection)	INbreast	YOLOv3
Su et al. [16]	2022	mAP: 0.650, F1: 0.745	CBIS-DDSM	YOLO-LOGO (YOLOv5L6)
Ribeiro et al. [42]	2022	mAP: 0.694, F1: 0.712	CBIS-DDSM	YOLOv5
Karaca Aydemir et al. [43]	2025	mAP: 0.843; F1: 0.812	INbreast (TL from CBIS-DDSM/VinDr-Mammo)	YOLOv5-CAD (YOLOv5)
Cao et al. [44]	2024	${\mathrm{mAP}}_{50}$: 0.873	INbreast	YOLOv8 with TL
Current study	2025	${\mathrm{mAP}}_{50}$: 0.995, F1: 0.984 (mass)	INbreast	YOLOv12-L with TL
Current study	2025	${\mathrm{mAP}}_{50}$: 0.697, F1: 0.708 (combined mass)	Combined dataset	RTMDet-X with TL

## Data Availability

The datasets used in this study are sourced from publicly available repositories or can be accessed upon request: INbreast is available at: https://www.kaggle.com/datasets/ramanathansp20/inbreast-dataset (accessed on 12 May 2025); CBIS-DDSM can be downloaded from The Cancer Imaging Archive (TCIA): https://www.cancerimagingarchive.net/collections/cbis-ddsm (accessed on 12 May 2025); RSNA Pneumonia Detection Challenge: https://www.kaggle.com/competitions/rsna-pneumonia-detection-challenge (accessed on 3 July 2025); ChestX-Det10: https://github.com/Deepwise-AILab/ChestX-Det10-Dataset (accessed on 3 July 2025); FracAtlas: https://figshare.com/articles/dataset/The_dataset/22363012?file=43283628 (accessed on 4 July 2025); COVID-19 Image Data Collection: https://github.com/ieee8023/covid-chestxray-dataset (accessed on 12 May 2025). Datasets available on request: VinDr-Mammo is accessible upon request at: https://vindr.ai/datasets/mammo (accessed on 12 May 2025); EMBED is available for academic use upon request through the responsible data custodians as described in the original publication: https://registry.opendata.aws/emory-breast-imaging-dataset-embed/ (accessed on 13 May 2025); VinDr-CXR: https://physionet.org/content/vindr-cxr/1.0.0/ (accessed on 2 July 2025); VinDr-SpineXR: https://physionet.org/content/vindr-spinexr/1.0.0/ (accessed on 4 July 2025).

## Abbreviations

The following abbreviations are used in this manuscript:

BI-RADS	Breast Imaging–Reporting and Data System
CAD	Computer-Aided Detection
CLAHE	Contrast Limited Adaptive Histogram Equalization
CNN	Convolutional Neural Network
CXR	Chest X-ray
DBT	Digital Breast Tomosynthesis
DDSM	Digital Database for Screening Mammography
DETR	DEtection TRansformer
FFDM	Full-Field Digital Mammography
FPN	Feature Pyramid Network
IoU	Intersection over Union
mAP	Mean Average Precision
NMS	Non-Maximum Suppression
ROC	Receiver Operating Characteristic
ROI	Region of Interest
SVM	Support Vector Machine
TL	Transfer Learning
YOLO	You Only Look Once
